# Supplementary material for: Prolyl dihydroxylation of unassembled uS12/Rps23 regulates fungal hypoxic adaptation
Source: eLife. 2017 Oct 30;6:e28563. doi: 10.7554/eLife.28563 (PMC5690285; doi:10.7554/eLife.28563)
Supplement: Supplementary file 1. — Genotypes and references for the yeast strains used in the study. [file elife-28563-supp1.docx]

**Supplemental Table 1. Yeast strains**

| **Strain** | **Genotype** | **Source** | **Figure(s)** |
| --- | --- | --- | --- |
| KGY425 | *h^-^ his3-D1 leu1-32 ura4-D18 ade6-M210* | ATCC | **1**D,F; **1S1**B; **3**A-D; **4**A; **5**B; **6**A-C; **6S1**A; **7**E,F |
| PEY1801 | *h^-^ his3-D1 leu1-32 ura4-D18 ade6-M210 ofd1Δ::natMX6* | this study | **1**D,F; **3**A-D; **4**B; **6**A,B; **6S1**A,B |
| PEY1152 | *h^-^ his3-D1 leu1-32 ura4-D18 ade6-M210 ofd1 H142A D144A* | Hughes and Espenshade, 2008 | **1**D,F |
| PEY1802 | *h^-^ his3-D1 leu1-32 ura4-D18 ade6-M210 nro1Δ::natMX6* | this study | **3**A-D; **4**A,B; **6**A,B |
| PEY1803 | *h^+^ his3-D1 leu1-32 ura4-D18 ade6-M210 rps2302Δ::natMX6* | this study | **3**A-D; **6**A-C; **1S1**B; **6S1**A |
| PEY1804 | *h^-^ his3-D1 leu1-32 ura4-D18 ade6-M210 rps23Δ::natMX6* | this study | **3**A-D; **6**A-C; **1S1**B; **6S1**A; **7S1**A |
| PEY1805 | *h^-^ his3-D1 leu1-32 ura4-D18 ade6-M210 rps2302^+^-GFP(S65T)-kanMX6* | this study | **5**A,B |
| PEY1806 | *h^-^ his3-D1 leu1-32 ura4-D18 ade6-M210 rps2302^+^-GFP(S65T)-kanMX6 nro1Δ::natMX6* | this study | **5**A,B |
| PEY1807 | *h^-^ his3-D1 leu1-32 ura4-D18 ade6-M210 mCherry-ofd1-T_ADH1_-P_URA4_-kan^r^-T_TEF_* | this study | **5**C |
| PEY1808 | *h^-^ his3-D1 leu1-32 ura4-D18 ade6-M210 rps23Δ::natMX6 mCherry-ofd1-T_ADH1_-P_URA4_-kan^r^-T_TEF_* | this study | **5**C |
| PEY1809 | *h^-^ his3-D1 leu1-32 ura4-D18 ade6-M210 nro1Δ::kanMX6 mCherry-ofd1-T_ADH1_-P_URA4_-kan^r^-T_TEF_* | this study | **5**C |
| PEY1811 | *h^-^ his3-D1 leu1-32 ura4-D18 ade6-M210 rps2302Δ::natMX6 sre1(aa1-440)* | this study | **6**D; **7**D; **7S1**C-D |
| PEY1812 | *h^-^ his3-D1 leu1-32 ura4-D18 ade6-M210 rps23Δ::natMX6 sre1(aa1-440)* | this study | **6**D |
| PEY1813 | *h^+^ his3-D1 leu1-32 ura4-D18 ade6-M210 ofd1Δ::kanMX6 rps23Δ::natMX6 sre1(aa1-440)* | this study | **6**D |
| PEY1814 | *h^+^ his3-D1 leu1-32 ura4-D18 ade6-M210 ofd1Δ::kanMX6 rps2302Δ::natMX6 sre1(aa1-440)* | this study | **6**D |
| PEY522 | *h^-^ his3-D1 leu1-32 ura4-D18 ade6-M210 sre1Δ::kanMX6* | Hughes et al., 2005 | **6**A-C; **6S1**A |
| ED666 | *h^+^ leu1-32 ura4-D18 ade6-M210* | Bioneer | **6S1**B |
| *rps23Δ* | *h^+^ ade6-M210 or ade6-M216 ura4-D18 leu1-32 rps23Δ::kanMX4* | Bioneer | **6S1**B |
| *rpl23Δ* | *h^+^ ade6-M210 or ade6-M216 ura4-D18 leu1-32 rpl23Δ::kanMX4* | Bioneer | **6S1**B |
| *sre1Δ* | *h^+^ ade6-M210 or ade6-M216 ura4-D18 leu1-32 sre1Δ::kanMX4* | Bioneer | **6S1**B |
| *rps25Δ* | *h^+^ ade6-M210 or ade6-M216 ura4-D18 leu1-32 rps25Δ::kanMX4* | Bioneer | **6S1**B |
| PEY1816 | *h^+^ his3-D1 leu1-32 ura4-D18 ade6-M210 GST-rps2302-T_ADH1_-P_URA4_-kan^r^-T_TEF_* | this study | **7S1**A |
| PEY1817 | *h^+^ his3-D1 leu1-32 ura4-D18 ade6-M210 rps2302^+^-T_ADH1_-P_URA4_-kan^r^-T_TEF_* | this study | **7S1**A |
| PEY873 | *h^-^ his3-D1 leu1-32 ura4-D18 ade6-M210 ofd1Δ::kanMX6 sre1(aa1-440)* | Hughes and Espenshade, 2008 | **6**D |
| PEY875 | *h^-^ his3-D1 leu1-32 ura4-D18 ade6-M210 sre1(aa1-440)* | Hughes and Espenshade, 2008 | **6**D; **7**C |
| PEY1290 | *h^+^ his-D1, leu1-32, ade6-M210, ura4-D18::2xSRE-ura4^+^-kanMX, sre1(aa1-440)* | Lee et al., 2009 | **7**B |
| PEY1489 | *h^+^ his-D1, leu1-32, ade6-M210, ura4-D18::2xSRE-ura4^+^-kanMX, sre1Δ::kanMX6* | this study | **7**B |
| AH109 | *MATα trp1-901 leu2-3,112 ura3-52 his3-200 gal4Δ gal80Δ LYS2∷GAL1_UAS_-GAL1_TATA_-HIS3 GAL2_UAS_-GAL2_TATA_-ADE2 URA3∷MEL1_UAS_-MEL_TATA_-lacZ* | James et al., 1996;   A. Holtz, unpublished | **1**A; **1S1**A |
| PEY1849 | *h^+^ his3-D1 leu1-32 ura4-D18 ade6-M210 rps2302 P62A-GFP(S65T)- T_ADH1_-P_URA4_-kan^r^-T_TEF_* | this study | **5**A,B |
| PEY1847 | *h^-^ his3-D1 leu1-32 ura4-D18 ade6-M210 rps2302^+^-GFP(S65T)-kanMX6 ofd1Δ::natMX6* | this study | **5**A,B |
| PEY1848 | *h^-^ his3-D1 leu1-32 ura4-D18 ade6-M210 rps2302^+^-GFP(S65T)-kanMX6 ofd1 H142A D144A* | this study | **5**A,B |
| PEY1410 | *h^-^ his3-D1 leu1-32 ura4-D18 ade6-M210 nro1Δ::kanMX4 sre1(aa1-440)* | Lee et al., 2009 | **6**D; **7**D; **7S1**B |
